# Alternative Sources of Omega-3 Fats: Can We Find a Sustainable Substitute for Fish?

**DOI:** 10.3390/nu5041301

**Published:** 2013-04-18

**Authors:** Georgia Lenihan-Geels, Karen S. Bishop, Lynnette R. Ferguson

**Affiliations:** 1 Discipline of Nutrition, Faculty of Medical & Health Sciences, University of Auckland, Private Bag 92019, Auckland, 1142, New Zealand; E-Mail: l.ferguson@auckland.ac.nz; 2 Auckland Cancer Society Research Center, Faculty of Medical & Health Sciences, University of Auckland, Private Bag 92019, Auckland, 1142, New Zealand; E-Mail: k.bishop@auckland.ac.nz; 3 Nutrigenomics New Zealand, University of Auckland, Private Bag 92019, Auckland, 1142, New Zealand

**Keywords:** eicosapentaenoic acid (EPA), docosahexaenoic acid (DHA), omega-3, inflammation, dietary fatty acids, fish oils, stearidonic acid, algae

## Abstract

Increasing demand for eicosapentaenoic acid (EPA) and docosahexaenoic acid (DHA) containing fish oils is putting pressure on fish species and numbers. Fisheries provide fish for human consumption, supplement production and fish feeds and are currently supplying fish at a maximum historical rate, suggesting mass-scale fishing is no longer sustainable. However, the health properties of EPA and DHA long-chain (LC) omega-3 polyunsaturated fatty acids (PUFA) demonstrate the necessity for these oils in our diets. EPA and DHA from fish oils show favourable effects in inflammatory bowel disease, some cancers and cardiovascular complications. The high prevalence of these diseases worldwide indicates the requirement for alternative sources of LC-PUFA. Strategies have included plant-based fish diets, although this may compromise the health benefits associated with fish oils. Alternatively, stearidonic acid, the product of α-linolenic acid desaturation, may act as an EPA-enhancing fatty acid. Additionally, algae oils may be a promising omega-3 PUFA source for the future. Algae are beneficial for multiple industries, offering a source of biodiesel and livestock feeds. However, further research is required to develop efficient and sustainable LC-PUFA production from algae. This paper summarises the recent research for developing prospective substitutes for omega-3 PUFA and the current limitations that are faced.

## 1. Introduction

**Figure 1:**
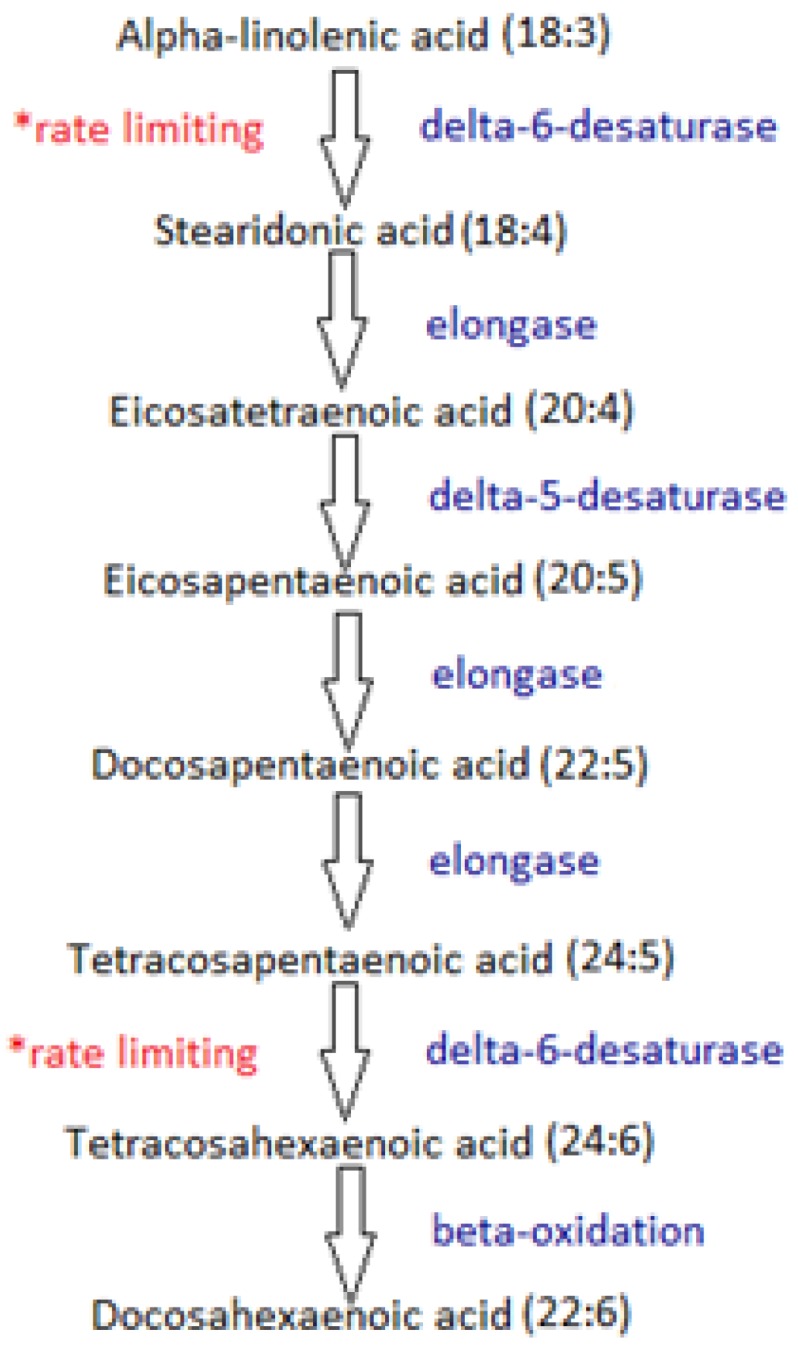
A series of elongation and desaturation reactions allows conversion of short-chain omega-3 fatty acids into the longer chain polyunsaturated fatty acids such as eicosapentaenoic acid (EPA) and docosahexaenoic acid (DHA). The delta-6-desaturase catalyses the rate-limiting enzymatic reaction, leading to inefficient conversion to SDA (stearidonic acid) in humans [6,7,8].

Fish consumption and omega-3 supplementation have attracted considerable interest in the past few decades in relation to their health benefits. Fish oils provide a source of eicosapentaenoic acid (EPA) and docosahexaenoic acid (DHA), two fatty acids now recognised as an important part of the human diet [1]. EPA and DHA are highly unsaturated fatty acids synthesised from alpha-linolenic acid (ALA) and other fatty acids in the omega-3 pathway (Figure 1). These long-chain fatty acids, comprising a chain length of at least 16 carbon atoms, have shown modulatory effects on the inflammatory pathway resulting in beneficial outcomes in the risk of inflammatory bowel disease (IBD), arthritis, cardiovascular disease and some cancers [1,2,3]. The major sources of these omega-3 fatty acids are oily fish species including salmon, mackerel and herring [4]. Fisheries are currently producing the maximum fish stocks per annum in order to supply fish for human consumption, as well as supplying feed for industrial fish farms and fish oil supplements, resulting in a substantial effect on fish levels and the possibility of extinction [5]. However, an expansive literature indicates that omega-3 fish oils are crucial dietary components. In order to protect fish species and the oceans’ ecosystems, alternative sources for long-chain polyunsaturated fatty acids (LC-PUFA) are required. Currently explored alternatives include plant oils with high omega-3 content, the use of stearidonic acid and algae oils.

This review highlights the importance of LC-PUFA in our diet, focusing on their role in inflammation and risk and progression of particular diseases. Additionally, possible alternative sources for these LC-PUFA are discussed.

## 2. Mechanisms of Fatty Acids in Inflammation

**Figure 2:**
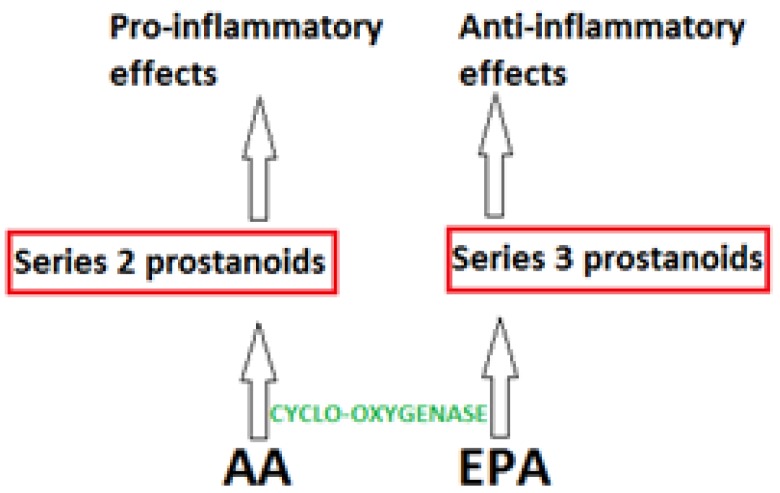
The synthesis of prostanoids is catalysed by cyclooxygenase enzymes. Cleavage of arachidonic acid (AA) and EPA by phospholipase A_2_ (not shown) allows free AA and EPA to be converted to pro-inflammatory and anti-inflammatory mediators, respectively [15].

Two major fates of dietary LC-PUFA include incorporation into cell plasma membranes as phospholipids and β-oxidation to produce energy. Following integration into cell membranes, phospholipids act to maintain membrane fluidity and act as precursors and signaling molecules for multiple pathways [9]. Following cleavage of membrane phospholipids by phospholipase A_2_, lipooxygenase (LOX) and cyclooxygenase (COX) enzymes act on free fatty acids to produce pro- or anti-inflammatory mediators [10] (Figure 2). Due to the dual role of the LOX and COX enzymes in converting omega-3 and omega-6 PUFA, the two classes of fatty acids show competition for these enzymes (Figure 2). This process occurs following an initial response with subsequent cleavage of a fatty acid from diacylglycerol, which also has signaling activity [11]. Therefore, the type of fatty acids present in the cell membrane have an effect on the mediators synthesised, influencing the net outcome during an inflammatory response.

Individuals with a high consumption of fish oils show a greater proportion of EPA and DHA-containing phospholipids in particular cell types, compared to individuals consuming plant oil supplements [12,13]. This in turn affects the levels of particular metabolites. Arachidonic acid (AA), an omega-6 fatty acid, acts as a precursor for the pro-inflammatory, series 2 prostanoids, whereas EPA provides precursors for the production of the anti-inflammatory mediator family of series 3 prostanoids (Figure 2). Although inflammation is a key process in the innate immune system, excessive production of pro-inflammatory products during chronic inflammation can have detrimental effects and increase susceptibility to disease. This occurs via an increase in reactive oxygen species, induction of a state of cellular stress, alteration in important bioactive molecules such as growth factors and remodeling of matrix proteins and tissue structure [14]. 

## 3. Inflammation, Omega-3 Pufa and Health Benefits

Chronic inflammation has shown an association with many of the current, prevalent diseases including cancer and inflammatory bowel disease. Although the co-occurrence of tumour formation and inflammation may arise from different sources, the two processes are often connected, indicating that inflammation has a recognised role in carcinogenesis [14]. Specifically, high levels of particular cytokines may influence overall survival in colorectal cancer patients [16]. In addition, immunohistochemical analyses of tissue biopsies from benign prostate cancer patients suggest a correlation between certain inflammatory markers, volume of prostatic tissue and progression of the disease [17]. With regards to inflammatory bowel disease, pro-inflammatory interleukin-23 (IL-23) is crucial for the development of T-cell mediated colitis in mice [18] and colitis may be reversed following treatment with a monoclonal anti-IL-23 [19]. Inflammation also has a well-known role in arthritis, atopic disease and cardiovascular disease [20,21,22]. Therefore, dietary factors such as omega-3 LC-PUFA that can modulate the inflammatory response are likely to have a significant contribution to risk and progression of these diseases.

As dietary fatty acids show a prominent role in the inflammatory response, researchers have investigated the relationship between consumption of particular fatty acids with inflammatory-related disease, such as cancer. Dietary omega-3 fatty acids, stearidonic acid (SDA) and EPA, have shown to reduce tumour necrosis factor-α (TNF-α) in whole blood, a well-known pro-inflammatory cytokine involved in carcinogenesis [23,24]. A recent paper outlines the effect of omega-3 fatty acids in retarding the progression of intraepithelial neoplasms to adenocarcinoma in the pancreas [25]. The study, carried out in mice, suggests that a higher proportion of omega-3 fatty acids in pancreatic cells leads to reduced progression of pancreatic duct tumours. However, in a recent meta-analysis no significant association was found between prostate cancer and omega-3 intake [26]. These discrepancies may indicate that a high ratio of these fatty acids may influence cancer risk [27]. Alternatively, a high ω-6/ω-3 ratio has suggested a non-significant relationship with colorectal cancer, indicating the importance of sex, race, genetics and the type of cancer in question [28,29]. In conclusion, a diet high in omega-6 but low in omega-3 fatty acids may increase the risk of some cancers [30,31]. This is due to competition for COX and LOX enzymes between omega-3 fatty acids and omega-6 fatty acids, thereby affecting the eicosanoids synthesized [14]. Therefore, increased consumption of long-chain omega-3 fatty acids may reduce risk of cancers via regulation of the inflammatory response. 

LC-PUFA-containing fish oils may also modulate diseases other than cancer. Benefits of *n*-3 LC-PUFA in cardiovascular disease include a reduction in fatal coronary events and sudden cardiac death [32]. Furthermore, major depressive disorder patients with low total *n*-3 and low EPA status correlated with lower survival rates following chronic heart failure [33]. The microvascular endothelium of the intestine has shown to respond to dietary fats in a rat model of intestinal colitis [34]. DHA significantly reduced expression of pro-inflammatory mediators including VCAM-1, IL-6 and COX-2. Additionally, there is a lower risk of Crohn’s disease in fish consumers [35]. These studies highlight a major contribution of dietary fatty acids in the modulation of inflammatory responses and the implication of this in disease outcome. 

## 4. Do Plant Oils Offer an Alternative to Fish Oils?

Plants high in omega-3 PUFA, such as linseed, primrose, echium and hempseed, contain only shorter-chain omega-3 PUFA and none, or low levels of EPA and DHA [36,37]. However, the ability to use plant oils in fish feed and human supplement production would substantially reduce the impact on fish levels, introducing a much more sustainable and economical source. Unfortunately, fishery studies have revealed that fish diets high in plant oils correlate with a lower accumulation of beneficial omega-3 fatty acids in fish flesh [38,39]. Capelin fish oil diets show lower levels of 18:2 (*n*-6) in belly flap and red muscle tissue relative to palm oil and sunflower oil diets in Atlantic salmon [39]. Similar results were demonstrated in total flesh lipids with linseed and rapeseed oil diets [38] signifying the importance of long-chain omega-3 fats in fish diets to maintain the nutritional quality of flesh. 

Seierstad *et al.* [40] observed the effect of consuming differently fed Atlantic salmon on markers of vascular inflammation and serum lipid profiles in patients with coronary heart disease (CHD). Fish were previously fed on 100% fish oil, 100% rapeseed oil or an equal combination, resulting in varying compositions of fatty acids between the three fish groups. Following a 6-week trial, patients consuming the fish oil-fed fish showed a significant increase in total omega-3 fatty acids and a higher ω-3/ω-6 ratio compared to baseline levels [40]. In the 100% rapeseed oil fish group, the ω-3/ω-6 ratio and levels of DHA were reduced in comparison to baseline. Changes in serum triacylglycerides were also observed with a significant decrease from baseline in the 100% fish oil-fed fish group. The fish oil-fed fish dietary group showed an increase in HDL lipoproteins, a lipoprotein known reduce plaque formation during atherogenesis [41]. In regards to inflammation, the fish oil group showed significantly lower levels of VCAM-1, interleukin-6 (IL-6) and TNF-α in comparison to baseline [40]. These inflammatory mediators contribute to pro-inflammatory effects and infiltration of neutrophils. Therefore, this study presents the possible effects associated with human consumption of fish fed on plant-based diets. Pro-inflammatory markers are reduced following consumption of fish fed fish oil feeds compared to plant oil diets, suggesting these fish are the most beneficial to human health and inflammation in CHD patients. 

Fish oil-finishing diets comprise a period of feeding on plant oils with a subsequent follow-up diet of fish oil. A 20-week fish oil-finishing diet in salmon that were previously fed either 100% fish oil, 100% rapeseed oil or 100% linseed oil for 50 weeks showed interesting results, wherein the fish on vegetable oil diets demonstrated levels of EPA and DHA up to 80% ensuing the follow-up, compared to those observed in solely fish oil-fed fish [38]. A similar study showed up to 88% restoration of EPA and DHA after switching Atlantic salmon from a 40-week linseed oil diet to a 24-week capelin oil diet [42]. A recent study investigated the effect of palm fatty acid distillate, a by-product of palm oil refining, as an effective preceding diet for Atlantic salmon fed on a short-term, fish oil-finishing diet [43]. The high proportion of free saturated fatty acids allows easy digestion and may be preferential substrates for β-oxidation, thereby reducing oxidation of long-chain polyunsaturated fatty acids (LC-PUFA). Furthermore, a short-term deprivation period between a preliminary vegetable oil diet and a fish oil follow-up diet demonstrated an increase in omega-3 deposition, notably DHA, at certain tissues including the fillet [43]. One drawback of the study design was the small size of the Atlantic salmon used. Fish at market size would likely require different diet and deprivation period durations. These studies indicate that a fish-oil finishing diet, initiated following a plant oil-based diet, may reduce the decline in flesh LC-PUFA.

## 5. Stearidonic Acid: Can We Bypass the Rate-Limiting Step?

SDA is synthesised in humans and plants following desaturation of ALA by the delta-6 desaturase, as demonstrated in the omega-3 pathway (Figure 1). Delta-6-desaturase is coded by a fatty acid desaturase (FADS) gene in humans and the conversion rates of this enzyme have been suggested as inefficient [7,44]. SDA exists in relatively low amounts in most plant oils. However, it is found in the *Boraginaceae* and *Primulaceae* families, commonly known as the borage and primrose plant families, as well as the *Cannabaceae* family [37]. Specifically, *Echium plantagineum* and *Buglossoides arvensis*, two species of *Boraginaceae*, showed the highest amount of SDA relative to total fatty acids, up to 12.5% and 20%, respectively [8,45]. It is important to note that levels and composition of fatty acids in plant seeds may vary widely in species in response to climate, soil, cultivation methods and growth stage of the plant [37]. Therefore, further experimentation would be required to optimise production of SDA and to determine the efficiency and sustainability of such a crop as a primary supplier of omega-3 SDA for human consumption. 

Due to the low conversion rate of ALA to EPA, researchers have posed the question as to whether supplying SDA may increase EPA levels at a more adequate level than ALA supplementation. The basis of this proposal lies in the ability to bypass the rate limiting enzyme, delta-6-desaturase, thereby enhancing production of EPA. Lemke *et al.* [46] suggest that SDA supplementation of 4.2 g a day, from SDA-enriched soybean oil for 12 weeks, enhances the EPA component of the omega-3 index, in red blood cells in comparison to regular soybean oil [46]. Comparable results were seen in subjects on a 1 g/day EPA supplement, suggesting EPA supplementation is around 4 times more efficient than SDA. Surette *et al.* [8] suggest triacylglyceride-lowering properties of SDA, as shown by echium oil supplementation [8]. This may be beneficial for those at risk of cardiovascular disease. A separate study demonstrated SDA ingestion was efficient in raising tissue EPA at higher levels than dietary ALA, although slightly less efficient than EPA supplementation [44]. It is important to note that to date no study on SDA has shown changes in DHA concentrations [44,46,47]. Therefore, alternative methods for increasing DHA might be required. A recent study tested the effect of dietary Ahiflower™ oil, derived from the *Buglossoides arvensis* plant, on fatty acid compositions of mice [45]. The reliable study design matched diets to mirror human diets, rendering results appropriate for human comparison. EPA and DPA compositions of liver and intestinal tissue increased following the Ahiflower™ diet [45]. The DHA composition of liver tissue also increased, whereas no significant differences in EPA or DHA levels in brain tissue were observed between the control and Ahiflower™ diets.

With regards to safety of SDA, rat studies suggest no adverse effects of daily SDA ingestion up to 600 mg per kg of body weight, signifying 1.9 g/day of SDA may be a safe dose for the average person of 60 kg [48]. Conversely, Lemke *et al.* [46] reported minor adverse events in human subjects taking 4.2 g/day SDA, including digestive disturbances and abdominal discomfort, with two subjects developing gastroenteritis [46]. As similar effects were observed in the control and EPA-diet group in addition to the SDA-diet group, it may be that the SDA is not the specific cause. Interestingly, it has been suggested that the ability of SDA to increase EPA tissue concentrations decreases following the optimum dose [49]. In addition, levels of EPA would likely depend on the delivery method. For example, SDA may be supplied as an oil capsule, or enriched into specific food products such as margarine. Food manufacturers must take this into consideration during development of SDA-containing food products. At this stage, further investigation, preferably in the form of human clinical trials, is required to determine the optimal dose.

### 5.1. The Relationship of SDA Supplementation with Health and Disease

Although the data indicates that SDA may act as an efficient precursor of EPA synthesis, the relationship of SDA supplementation with biological outcomes must be specifically measured. It is therefore necessary to investigate downstream effects of SDA supplementation on inflammatory markers, blood lipid profiles, atherosclerotic plaque growth and changes in gene expression to determine the true role of SDA consumption in prevention and progression of diseases such as diabetes mellitus, cancer and coronary heart disease. From this, individuals at risk of disease may turn to SDA supplementation as an alternative to fish oils. Studies on fish oils and the outcome of disease are based on the natural composition of fish oil, which generally contains both EPA and DHA [50]. Therefore, as SDA is thought to increase tissue EPA only, studies involving SDA intake must specifically monitor risk and outcome of disease in order to claim health benefits. A recent review outlines the current lack of data on the beneficial biological effects of SDA [51]. Long-term, prospective studies in humans are yet to be carried out, however some animal studies suggest advantageous effects on disease biomarkers, as outlined herein. 

Recently, a reduction in plasma cholesterol, most notably low-density lipoprotein (LDL) and very-low density lipoprotein (VLDL), and triacylglycerides was observed in mice on an echium oil supplement in comparison to palm oil-fed mice over a 12-week period [52]. Furthermore, the effect of the SDA-containing echium oil almost mirrored that of fish oil. The levels of these lipid constituents are known to correlate with risk of atherosclerosis and heart disease [53]. This study specifically analysed aortic lesion surface area to monitor the changes in plaque size following each of the 12-week diets. Both aortic lesion surface area and aortic cholesterol levels were lowered significantly in echium oil and fish oil-fed mice [52]. This suggests that supplementation of echium or fish oil over 12 weeks may directly reduce risk of atherosclerotic growth in mice. Additional research is required to underline the exact processes leading to a reduction in these parameters.

Banz *et al.* [54] have suggested a potential role of SDA in reducing risk of diabetes mellitus. Although more studies are required to assess this theory, the observed effects of SDA reducing inflammation, lowering prostaglandin E2 synthesis and reducing blood triacylglycerides signify a reduction in diabetes mellitus biomarkers [8,13,55]. Changes in gene expression are also important factors to consider. A pig study showed many changes in expression of genes following a 35-day SDA diet [56]. The stearoyl-CoA desaturase gene involved in fatty acid synthesis was down regulated along with genes involved in diacylglycerol synthesis. Interestingly, PON3, a gene coding the paraoxonase 3 protein thought to inhibit inflammation and LDL oxidation, was up-regulated, while pro-inflammatory C-reactive protein was down-regulated, suggesting an SDA diet of 3.7 g/day in humans may have beneficial effects in atherosclerosis development [56].

**Figure 3:**
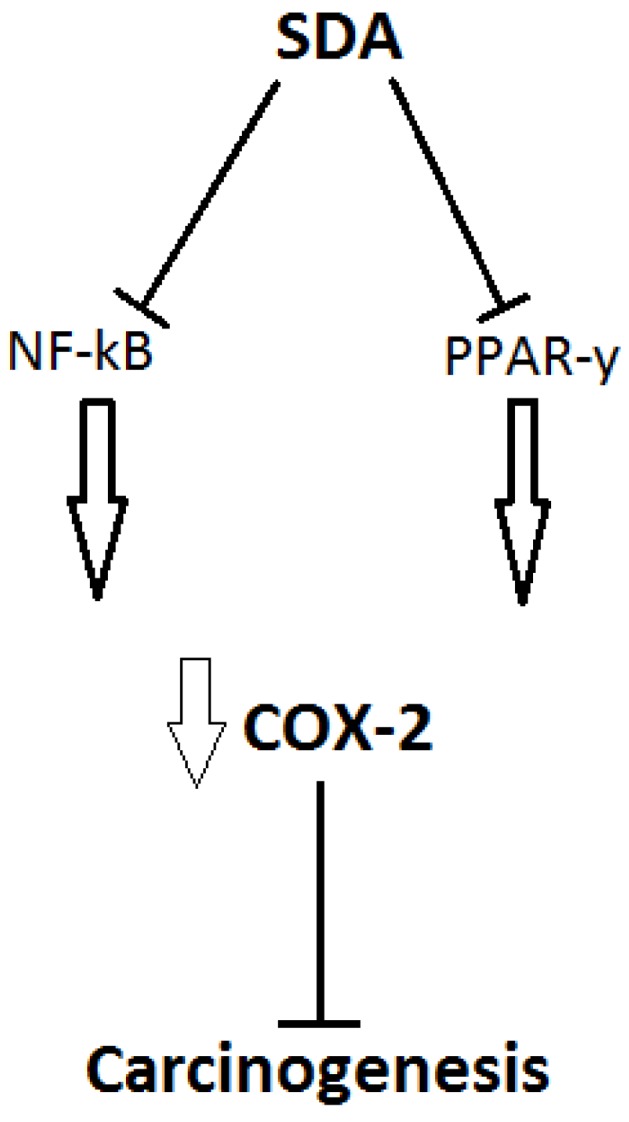
SDA has shown potential as a NF-κB and PPAR-γ reducing metabolite, leading to reduced COX-2 transcription, a protein seen in high amounts in some tumours [52]. Figure constructed with data from [51,52].

Horia and Watkins [57] demonstrated a protective role of SDA *versus* ALA in MDA-MB-231 cells, a breast cancer cell line. This included better efficiency at reducing COX-2 transcription and translation via reduction in two transcription factors, nuclear factor kappa-light-chain-enhancer of activated B cells (NFκB) and peroxisome-proliferator activated receptor-γ (PPAR-γ) (Figure 3). COX-2 is responsible for production of inflammatory mediators and high levels of this enzyme have been observed in some tumours [58]. Furthermore, as SDA raises tissue EPA and not DHA, it suggests the beneficial effects of SDA in relation to carcinogenesis are irrespective of DHA. Additionally, SDA-supplemented mice showed much smaller tumour size recurrence compared to linoleic acid (LA) [59].

### 5.2. Can SDA Substitute as an Animal Feed?

An alternative to direct SDA supplementation as a potential method for increasing LC omega-3 PUFA in the human diet is to feed fish, poultry and other livestock with high-SDA containing oils. However, it is important to note that this approach excludes vegetarians. Nonetheless, a recent study suggests echium oil may increase poultry tissue omega-3 content [60]. Breast and thigh muscle tissue evaluation signified an increase in all fatty acids of the omega-3 pathway, excluding DHA in the thigh, when compared to muscle tissues of chickens fed the rapeseed oil diet. This resulted in a higher total omega-3 content across both thigh and breast muscle following the echium oil diet [60]. This study signifies that echium oil may act as an effective dietary component of poultry in order to increase omega-3 fatty acids, excluding DHA, and therefore may increase health benefits of chicken meat. 

Some studies suggest no improvement in EPA content of fish or lamb flesh following an SDA-rich oil diet [61,62,63]. Although no increase in EPA was observed in red or white muscle of Atlantic salmon, an increase in DHA, although still at minimal amounts, was found in both groups fed SDA and fish oil, in comparison to canola oil-fed fish [62]. Additionally, this study suggested higher metabolic activities of the omega-3 pathway when fish were fed SDA *versus* fish oil. This suggests different oil compositions in fish diets may affect the activity of enzymes involved in the omega-3 pathway. Notably, researchers must acknowledge the developmental stage of fish being tested (pre- or post-smolt), as differences in metabolic activity are apparent [62].

A similar study in Rainbow trout suggested that echium oil is similar to linseed oil in its effects to raise EPA and DHA content of flesh, which is much less efficient than a fish oil diet [61]. The two main desaturases, delta-6 and delta-5 desaturase, showed differing activities in regards to the type of diet consumed and the fatty acid being desaturated [61]. This should be further investigated in fish, as it will allow better understanding of the fatty acid desaturation and elongation pathways in fatty fish. Furthermore, Kitessa *et al.* [63] presented data to suggest echium oil has no advantage over linseed oil in enhancing long chain omega-3 fatty acids in lamb tissues. Importantly, this study matched the different oils with regards to amount of precursor *n*-3 fatty acids, thereby balancing the intake of omega-3 fatty acids between the linseed and echium oil diets. The previous study showing higher EPA in echium oil-fed chickens compared to rapeseed oil-fed chickens may indeed be a result of higher omega-3 precursor content in the echium diet *versus* control diet. Additional studies taking the approach of Kitessa *et al.* [63] are required to further validate this theory. 

### 5.3. The Differential Effects of EPA and DHA in Inflammation

Following the finding that SDA may increase EPA levels more efficiently than ALA, it is important to note the individual effects of EPA, as most studies focus on a combination of EPA and DHA as they are found in fish oils [44,46,47]. Although both EPA and DHA have been shown to reduce interferon-γ and interleukin-2 (IL-2) in Jurkat cells, only EPA correlates with a reduction in IL-10 [64]. This is interesting as IL-10 is associated with anti-inflammatory properties [65]. These inflammatory mediators are associated with reduced inflammatory states when in abundance [14]. Furthermore, Weldon *et al.* [66] suggest DHA has higher potency for reducing IL-1β and IL-6, although both EPA and DHA showed similar effects on TNF-α levels. IL-1β has a role in systemic inflammatory states [67] whereas IL-6 levels rise rapidly during acute inflammation [68]. DHA also showed stronger inhibition of NF-κB, a pro-inflammatory transcription factor [66]. These studies highlight the potential differences between DHA and EPA in regulation of inflammation and this is important to consider when evaluating the health benefits of SDA, an EPA-raising omega-3 fatty acid. 

## 6. Algal Oils as a Source of EPA and DHA

Algae are the primary producers of the oceans’ ecosystems, providing the foundation of the oceanic food chain. Specifically, algae synthesise omega-3 fatty acids that are subsequently consumed by other marine life. Algae-derived oils are vegetarian-friendly and easy to grow on a large scale due to their small size. Superfluous lipid and protein during algal growth may be used as biodiesel and biomass for oil sources and animal feed, respectively [69,70]. This highlights the sustainable benefits of algae and the many potential gains from creating algal biofactories. 

There is an extensive number of algal species and each shows variability in the synthesis of EPA and DHA [71,72]. *Schizochytrium* sp., a heterotrophic thraustochytrid, produces elevated quantities of DHA and minimal levels of EPA [71]. *Schizochytrium* sp. is currently used in commercial products including infant formulas, food additives, cosmetic and pharmaceutical products [73]. One study investigating the thraustochytrid *Thraustochytrium* sp. demonstrated high DHA synthesis of up to 35% total fatty acids. Importantly, low nitrogen conditions with high supply of monosodium glutamate and yeast extract yielded the best growth and synthesis of fatty acids, indicating the importance of appropriate conditions. Other beneficial aspects of this strain include tolerance to high sodium chloride concentrations and production of several carotenoids [73]. Additional studies have analysed *Cryptocodinium cohnii*, another high-DHA synthesising microalgae, and such oils are also used in commercial products [72]. Conversely to *Schizochytrium* sp., *Cryptocodinium* sp. and other autotrophs and mixotrophs can fix carbon dioxide, indicating cost-efficiency and sustainability. 

Cost, extraction and purification methods are currently limiting the potential of using micro algal oils on a larger-scale [69]. Furthermore, additional experimentation to ensure optimal growth conditions for enhancing lipid biosynthesis, ideal species selection, quality control and sufficient methods for maximising ingestion and digestibility will aid the potential of algae oil as a major source of omega-3 fatty acids in our diet. 

## 7. Conclusions

In conclusion, omega-3 fatty acids possess highly beneficial effects for modulating risk of prevalent diseases. Due to the decline in fish species and number, alternative sources of these fatty acids are required. Potential substitutes include plant oils, stearidonic acid and algae oils. Plant oils contain low amounts of LC-PUFA and the use of plant oils in fish feed has shown mostly disappointing results. SDA has potential as an EPA-enhancing fatty acid, by bypassing the rate limiting enzyme delta-6-desaturase, although sources of SDA are limited. Lastly, algae offer a promising alternative. The use of algal oils will benefit multiple industries. Additional research into algal species, growth and lipid synthesis will enable this market to expand. 
